# Boron neutron capture therapy (BNCT) for experimental bladder cancer: systemic or intravesical approach

**DOI:** 10.1038/s41416-026-03418-w

**Published:** 2026-04-17

**Authors:** Kerem Teke, Cüneyt Özer, Büşra Yaprak Bayrak, İskender Atilla Reyhancan, Çiğdem Vural, Murat Kasap, Ayşegül Ünal Karabey, Gürler Akpınar, Efe Bosnalı, Neslihan Koyuncu, İbrahim Erkut Avcı, Onur Erbay, Muhammet Sahip Kızıltaş, Fatih Hunç, Zeyneb Camtakan, Önder Kara, Görkem Aksu, Özdal Dillioğlugil

**Affiliations:** 1 Department of Urology, Kocaeli University School of Medicine, Kocaeli, Turkey; 2 Department of Experimental Animal Laboratory, Kocaeli University School of Medicine, Kocaeli, Turkey; 3 Department of Pathology, Kocaeli University School of Medicine, Kocaeli, Turkey; 4 Energy Institutes, Istanbul Technical University, Istanbul, Turkey; 5 Department of Medical Biology, Kocaeli University School of Medicine, Kocaeli, Turkey; 6 Department of Radiation Oncology, Kocaeli University School of Medicine, Kocaeli, Turkey; 7 Department of Biochemistry, Kocaeli University School of Medicine, Kocaeli, Turkey

**Keywords:** Bladder cancer, Bladder cancer

## Abstract

**Background:**

To investigate the antitumoral and pro-inflammatory effect of Boron Neutron Capture Therapy (BNCT) following systemic or intravesical borophenilalanine administration, and to compare it with conventional radiotherapy (cRT) in an experimental bladder cancer (BC) model.

**Methods:**

Sixty-four Wistar rats were used, of which half were exposed to carcinogen to induce BC. Radiation therapy (RT) was performed both in the MARK TRIGA-II reactor for BNCT and in the Radiation Oncology divison for cRT. After necropsy, bladder and perivesical tissues were collected. Tumour staging, tumour burden, proliferative, and apoptotic indexes were evaluated for bladder samples. Bladder and perivesical tissues (colon, uterus, and anterior abdominal wall) were also assessed for inflammatory changes in H&E-stained sections, and also bladder TNF-α expressions was examined by immunohistochemistry and Western blot.

**Results:**

The animals with cancer had a 15–30% decrease in tumour burden after RT. The incidence of persistent papillary urothelial carcinoma was 100% in the Cancer-cRT group and 87.5% in the Cancer-BNCT-Sys group, whereas a lower incidence was observed in the Cancer-BNCT-IV group (71.4%). A lower proliferative and a higher apoptotic indexes were observed in  Cancer-BNCT-IV group compared to Cancer-cRT. Immunohistochemistry and Western blot for TNF-α expression showed that pro-inflammatory response in bladder was lower in BNCT-IV group among cancer treated groups. Moreover, there were lower proinflammatory adverse findings in perivesical tissues, including colon and uterus, in animals receiving BNCT-IV.

**Conclusion:**

Similar to cRT, systemic or intravesical BNCT resulted in a partial decrease in tumour burden, but fewer adverse findings by intravesical borophenilalanine.

## Introduction

Bladder cancer is the seventh most common cancer globally and is one of the types of cancer with high mortality and morbidity [1]. Bladder cancer is categorised as localised (sub-divided into muscle invasive and non-muscle invasive) and metastatic. Radical cystectomy or chemoradiotherapy is recommended, especially for the treatment of non-metastatic muscle invasive disease. The European Urology Guidelines report that, although, the outcome of radiotherapy in non-muscle invasive bladder cancer is not clear, there is evidence of the positive effect of radiotherapy on oncological outcomes, especially in BCG-nonresponsive disease and in high-risk non-muscle invasive bladder cancer [2].

Radiotherapy is often combined with transurethral resection and chemotherapy in muscle invasive bladder tumours, forming a multimodal treatment process and requires a multidisciplinary approach [3]. Although radiotherapy has antitumoral activity, complications may include difficulty with urination due to the inflammatory side effects in the bladder and inflammation and adhesions in the perivesical tissues. Therefore, the risk of morbidity due to radiotherapy is increased, and salvage cystectomy after radiotherapy may even become quite complicated [4].

Minimising vesical and perivesical side effects and confining the effect of radiotherapy within the tumour may be the optimal, and currently unique, treatment approach in the multimodal treatment of bladder cancer. For further clarification of this aim, it was decided to investigate the utility of a fusion reaction, called Boron Neutron Capture Therapy (BNCT), in which a neutron beam is captured by a boron molecule and converted into helium and lithium atoms, creating linear high energy at the cellular level in bladder cancer. This nuclear reaction causes DNA damage by releasing high linear energy at a distance of approximately 5-9 µm within cells in which boron has accumulated [5].

There is no previous study evaluating BNCT in orthotopic bladder cancer. The aim of this study was to investigate the antitumoral and inflammatory effect profiles of BNCT following systemic or intravesical Boron administration in comparison with conventional radiotherapy (cRT).

## Material and methods

### Experimental design

Total of 64 female Wistar rats were used in the experiments. Ethics committee approval was obtained for the study from Kocaeli University Animal Research Committee (KOU HADYEK, Decision No: 2/3-2020). To induce urothelial carcinogenesis, half of the rats were exposed to N-butyl-N-(4-hydroxybutyl) nitrosamine (OH-BBN) in drinking water (0.05%, w/v) for 16 weeks. The remainder did not receive carcinogen over a corresponding expermental period. Four rats that had and four that had not been exposed to OH-BBN were randomly chosen for B^10^ (boron) analysis after systemic (sys) and intravesical (IV) borophenylalanine (BPA) administration at week 22. The remaining 56 rats, half of which had been exposed to OH-BBN, were separated into groups as follows. For those not exposed to OH-BBN (*n* = 28): control-only (*n* = 4); control-cRT (*n* = 8); control-BNCT-sys (*n* = 8); and control-BNCT-IV (*n* = 8). For the animals exposed to OH-BBN: cancer-only (*n* = 4); cancer-cRT (*n* = 8); cancer-BNCT-sys (*n* = 8); and cancer-BNCT-IV (*n* = 8). The single fraction cRT or BNCT was performed for all appropriate groups, except for the cancer-only and control-only groups, at week 23 (Experimental schedule shown in Supplementary Fig. 1). Intraperitoneal (systemic) anaesthesia using ketamine (Ketalar^®^, Pfizer) and xylazine (Alfasan^®^, Woerden, Netherlands) was performed to immobilise animals prior to procedures, including intravesical instillation, neutron irradiation, and cRT.

### BPA preparation and administation

BPA (4-borono-l-phenylalanine; Sigma-Aldrich) was prepared and adminisitrated by two different methods for systemic and intravesical administration. Since a 250 mg/kg dose has been prescribed in clinical studies [6], this amount was also used in the current experiment. BPA is not easily soluble, so fructose was added to the solution and then the pH was adjusted to increase the BPA solubility, as previously described [7].

For systemic administration, 320 mg BPA was dissolved in the presence of 1.1 g fructose, in 7 mL PBS and 500 µL 5 N NaOH, with a total volume of 8.5 mL. The pH was adjusted to 7.6 by adding 60 µL 12 M HCl. For the animals receiving systemic BPA, the solution at a BPA concentration of 37.65 mg/mL was administered intraperitoneally (i.p.) to achieve a dose of 250 mg/kg at 90 min prior to neutron irradiation.

In order to prepare BPA formulation for intravesical administration, Dimethyl sulfoxide (DMSO) was used, which has been shown in previous studies to increase epithelial absorption with intravesical use, in addition to its high solvent and anti-inflammatory properties [8–10]. DMSO was used as the intravesical vehicle/solvent for BPA administration. This choice was based on experimental evidence demonstrating that intravesical DMSO markedly enhances transmural drug absorption across the urothelium and deeper layers of the bladder wall. In a well-established rat model, Yaman et al. showed that concomitant intravesical administration of DMSO enabled uniform penetration of epirubicin throughout the entire bladder wall, including papillary and hyperplastic lesions, whereas epirubicin without DMSO remained largely confined to superficial urothelial layers. These findings support the use of DMSO as an effective intravesical penetration enhancer but also indicate its potential to modify local tissue responses, including inflammatory signalling [8, 10]. Due to an expected bladder capacity in rats of approximately 1 mL, it was planned to obtain a similar intravesical concentration, since there was close to 40 mg BPA in 1 mL concentration of the systemically applied solution. For the intravesical formulation, 120 mg BPA was dissolved in the presence of 300 mg fructose, in 1.05 mL DMSO (99%) and 2.1 mL PBS in the presence of 200 µL NaOH (5 N) to obtain a total volume of 4 mL. The pH was adjusted to 7.6 with 80 µL HCl (12 M). The protocol was to administer 1 mL (40 mg/mL) of the prepared solution intravesically into the rat bladder. After anaesthetic induction, rat’s bladder was emptied initially and the solution was instilled into rat bladder 40 min prior to neutron irradiation using a 22 G angiocatheter.

### Boron assay in bladder tissue samples

As mentioned in the *Experimental Design* (Supplementary Fig. 1), four animals exposed to OH-BBN and four animals not exposed to OH-BBN at week 22 were used for a boron assay. Systemic BPA (250 mg/kg dose) was given to four animals, two of which had been exposed to OH-BBN. For intravesical administration, 40 mg BPA in 1 mL solution was instilled intravesically to four animals, two of which had been exposed to OH-BBN, after i.p. anaesthesia, and subsequently the catheter was clamped to ensure that the solution remains in the bladder until sacrification. Animals were sacrificed 90 min after systemic administration and 40 min after intravesical administration, and their bladders were removed. The bladders were opened vertically from the dome to the neck, and the lumen was washed with distilled water for 1 min. Then, bladder tissues were transferred to the İZAYDAŞ facility, a Kocaeli Metropolitan Municipality institution, at +4 °C. The boron accumulated in the bladder tissue was measured using inductively coupled plasma-mass spectrometry (ICP/MS), as previously described [11].

### Neutron irradiation for BNCT

- *Neutron source and supply:* the radial irradiation station located in the TRIGA Mark II Reactor at Istanbul Technical University was used as the source for the neutron beam for BNCT. A newly designed and manufactured neutron collimator and moderator system was placed in this port (Supplementary Fig. 2A-B). The neutron output of this collimator is 2 cm in diameter and is designed to deliver a neutron beam to the entire bladder region (Supplementary Fig. 3). Since the neutron spectrum of the reactor core consists of thermal, epithermal and fast neutron energy groups, a moderator was designed in the collimator to increase the number of thermal neutrons from these energy groups, i.e., the flux. In addition, high flux and high energy (average 7 MeV) gamma rays are emitted from the reactor. In order to reduce these gamma rays, cylindrical lead blocks were mounted on both sides of the collimator. This system was placed in the radial port of the reactor, and due to the high radiation level during neutron irradiation, an irradiation chamber consisting of 60 cm thick boron-containing concrete blocks was installed around the station. Thermal neutron flux was measured as 4 × 10^5 ^cm^−2^ s^−2^ from the two-cm diameter collimator outlet using 99.99% pure gold foil. Thermoluminescence dosimeters were used to determine how much gamma radiation the animals were exposed to. The average measured gamma ray equivalent dose values were 2077.37 ± 157.07 microSv/hour.
- *Animal irradiation process and set-up:* After the animals were sedated, optimal stabilisation was performed and they were placed in the animal fixation system in the irradiation room. The elapsed time for animal stabilisation after anaesthesia induction and then placement in the rat fixation system was approximately 10 min. Anaesthesia induction was performed 80 min after systemic BPA administration to the animals in the Control+BNCT-Sys and Cancer+BNCT-Sys groups. In the intravesical approach groups (Control+BNCT-IV and Cancer+BNCT-IV groups) transurethral BPA was instilled into the bladders and retained by clamping for 30 min. After being anaesthetised, there was a pause to allow boron absorbtion and dispersion in the bladder wall. In order to fix the animal for BNCT, it was placed in a nest in a polyethylene case at a right angle to the ground (Supplementary Fig. 4A-C). The animal, whose bladder area had been marked by tomography before, was aligned so that the lower quadrant of the abdomen coincided with the two cm diameter opening of the collimator. Then, each animal was exposed to 1 hour of neutron irradiation. Each animal was monitored in real-time by camera during the irradiation. The reactor was operated at full power (250 kW) under the required operating conditions. Fifteen minutes after the end of the neutron irradiation, the animal was taken and the next animal was placed. The procedures were repeated in order within the framework of the determined protocol. Neutron irradiation was applied to four animals each day for a total of eight days.

### Conventional radiotherapy

Rats from Control+cRT and Cancer+cRT groups were irradiated at the Kocaeli University Radiation Oncology Department at week 23. First, all rats were simulated in the supine position with a full bladder. Computed tomography (CT) simulation scan was performed using a Siemens Definition AS (Siemens Healthcare, Erlangen, Germany) CT device with a slice thickness of 1 mm. CT images confirmed that the posterior wall of the rat bladder was approximately 1–2 cm deep from the anterior abdominal wall, and the anterior wall of bladder was less than 0.5 cm deep (Supplementary Fig. 10A-D). The target volume was defined according to the report No. 62 of the International Radiation on Units Commission. The clinical target volume (CTV) was contoured by the radiation oncologist and a 0.5 cm planned target volume (PTV) margin was given [12]. A three dimensional conventional radiotherapy (3D-cRT) plan was created for each rat in the Eclipse V13.6 (Varian Medical Systems. Palo Alto, CA, USA) treatment planning system. The 3D-cRT technique was planned as a single field with 6 MV X-rays at a 0-degree gantry angle. The prescription dose was 600 cGy [13, 14]. The PTV was normalised to cover 95% of the prescribed dose. Since the bladder is close to the skin in the created treatment plans, a bolus of 0.5 cm tissue equivalent material was placed perpendicular to the irradiation surface in all treatment areas to draw the dose to the skin [15]. Prior to the treatment, kV and cone beam CT images of the rats were taken with the Varian Trilogy linear accelerator device (Varian Medical Systems. Palo Alto, CA, USA). The treatment isocenter was determined with image guidance and the treatment was applied (Supplementary Fig. 6A-C). The irradiation time varied between 1 and 1.5 min.

### Sacrifice protocol, collection of vesical and perivesical tissues, and tumour burden measurement

The rats were scheduled to be sacrificed using the exsanguination method consecutively between the 25th and 28th weeks. One animal each from the Control and Cancer groups, and two animals each from the Control+cRT, Cancer+cRT, Control+BNCT-Sys, and Cancer+BNCT-IV groups were sacrificed during every sacrifice week. One animal from the Control+BNCT-Sys group and one from the Cancer+BNCT-IV group was excluded from the study because they died prior to the designated sacrifice weeks. Therefore, two animals each from the Cancer+BNCT-IV and Control+BNCT-Sys groups were sacrificed between the 25th and 27th weeks (for a total 12 animals), and one animal each from these two groups was sacrificed at the 28th week.

After sacrifice, the animals’ bladders were removed, perivesical fatty tissues were separated, and then the bladders were weighed with a precision scale to assess tumour burden. Thereafter, the bladders were divided vertically from the dome to the neck into two equal parts, and one part selected randomly was placed in 10% formalin for histopathological evaluation. The remaining part was stored at −80 °C for Western blotting.

After the bladders were removed, the uterus, the colon next to the posterior part of the bladder, and the skinless anterior abdominal walls of the animals were placed in 10% formalin solution for histopathological evaluation of inflammation due to radiation therapy.

### Histopathological examination, Immunohistochemisty and Inflammation analysis

Half bladder sagittal sections were embedded in paraffin and sectioned at 5 µm thickness for histopathological (light microscopy after hematoxylin and eosin [H&E] staining) and immunohistochemistry (IHC), including Ki-67 proliferative index and TNF-α expression assessment. Following H&E staining, the slides were evaluated in accordance with the 2016 World Health Organisation (WHO) classification of bladder tumours.

Cellular proliferation was assessed using Ki-67 (SP6 Rabbit Monoclonal Antibody, Cell Marque™). The most densely stained area of Ki-67 expression was identified under the microscope, and the percentage of positively stained nuclei among 100 counted cells was calculated to determine the proliferative index.

Apoptotic activity was assessed using the TUNEL (Terminal deoxynucleotidyl transferase dUTP nick end labelling) method, which detects DNA fragmentation [16].

Proinflammatory response was evaluated by TNF-α immunostaining (Anti-TNF-α Antibody, clone 4E1, SC-130349, Santa Cruz Biotechnology, Dallas, TX, USA). TNF-α expression was quantified by counting the number of positive inflammatory cells in a single high-power field (HPF) within the most intensely stained region.

H&E staining was also performed on uterus, colon, and anterior abdominal wall tissues for inflammation assessment. The uterus was sectioned in the sagittal plane following a vertical cut, the posterior colon was sectioned axially, and the anterior abdominal wall was cut vertically along the midline and embedded in the sagittal plane. Bladder sections used for tumour evaluation were also included in the inflammation scoring.

Inflammation severity was graded based on the number of inflammatory cells in areas with high cellular infiltration: no to mild inflammation (0– < 50 cells); moderate to severe inflammation ( ≥ 50 cells).

All histopathological and immunohistochemical evaluations were performed in a blinded manner by two independent pathologists (BYB and CV).

### Western blotting

Bladder tissues were washed with cold buffer (250 mM sucrose, 25 mM Tris, pH 7.4), minced, and lysed in 2DE rehydration buffer (7 M urea, 2 M thiourea, 4% CHAPS, 30 mM Tris, pH 8.5, with protease inhibitors). Homogenisation was performed with stainless steel beads using a beadbeater. After sequential centrifugation (10,000 × rpm, 15 min; 15,000 × rpm, 30 min; both at 4 °C), supernatants were collected. Protein concentrations were determined by Bradford assay, and samples were stored at −80 °C. Pooled samples (100 µg per group) were re-quantified before analysis.

For Western blotting, 40 µg of protein was separated on 10% SDS-PAGE, transferred to nitrocellulose membranes, and blocked with 5% milk in TBS-T. Membranes were incubated overnight at 4 °C with anti-TNFα (1:1000, Santa Cruz Biotechnology, Cat # sc-52746), followed by secondary antibody (1:10,000) for 1 h at room temperature. Signals were visualised using electrochemiluminescence, and β-actin was used as the loading control. Experiments were performed in duplicate [17].

### Statistics

All statistical analyses were performed using IBM SPSS Statistics for Windows, Version 24.0 (IBM Corp., Armonk, NY, USA). Prior to group comparisons, the assumption of homogeneity of variances was assessed using Levene’s test. Depending on the design and structure of the data, either one-way or two-way analysis of variance (ANOVA) was applied. Two-way ANOVA was used to evaluate the main and interaction effects of urothelial carcinogenesis induction and treatment modality on continuous outcome variables such as bladder weight, Ki-67 proliferative index, apoptotic index, and TNF-α expression levels. For within-group comparisons, one-way ANOVA was conducted separately in carcinogenesis-induced and control groups to assess treatment-related differences. When a statistically significant main effect was identified, post hoc pairwise comparisons were performed using the Bonferroni test. In cases where the assumption of homogeneity of variances was violated (Levene’s test, *p* < 0.05), Welch’s ANOVA was used as a robust alternative. A two-sided *p*-value less than 0.05 was considered statistically significant throughout the analysis.

## Results

### B ^10^ analysis using ICP/MS

Prior to neutron irradiation, ICP/MS analysis demonstrated significantly higher boron levels in bladder tissues from tumour-bearing animals than in non–tumour-bearing (carcinogen-unexposed) controls after both systemic (4.39 ppm vs 1.51 ppm) and intravesical administration (10.89 ppm vs 1.82 ppm) (*p* < 0.05). Notably, intravesical delivery produced a substantially larger tumour-to-non-tumour boron concentration gradient ( ~ 6.0 fold for intravesical approach; ~2.9 fold systemic approach), supporting an improved local targeting advantage for BNCT-IV.

### Bladder cancer related macroscopic findings, tumour burden, and histopathological examination of bladder

Prior to necropsy, animals from cancer groups commonly had macroscopic haematuria (Supplementary Fig. 7). Throughout the sacrifice process, it was observed that the weight of bladders from animals with tumour induction was significantly greater than the weight of bladders from controls (F [1,46] = 102.78, *p* < 0.001, η² = 0.691). In control groups (Control, Control-cRT, Control-BNCT-Sys, Control-BNCT-IV), bladder weights did not differ significantly across treatment groups (F [3,23] = 1.43, *p* = 0.260). In contrast, in urothelial carcinogenesis induced groups (Cancer, Cancer-cRT, Cancer-BNCT-Sys, Cancer-BNCT-IV), a significant difference in bladder weight was detected (F [3,23]) = 3.44, *p* = 0.033). Post hoc analysis revealed that Cancer-BNCT-Sys significantly reduced bladder weight compared to the Cancer-only group (mean difference = 54.38 mg, *p* = 0.027). No other pairwise comparisons reached statistical significance (*p* > 0.05, Table 1). When compared to the Cancer-only group, the animals in the treatment groups exhibited diminished tumour burden, ranging from 15% to 30%. Macroscopic view of the luminal surface of an opened bladder from each experimental group is shown in Supplementary Fig. 8.

**Table 1:** Comparative data of bladder weights, precancerous or cancerous lesions, TNF -α expressions and proliferative-apoptotic index in the experimental groups.

	Control (*n* = *4*)	Control-cRT (*n* = 8)	Control-BNCT-Sys (*n* = 7)	Control-BNCT-IV (*n* = 8)	Cancer (*n* = 4)	Cancer-cRT (*n* = 8)	Cancer-BNCT-Sys (*n* = 8)	Cancer-BNCT-IV (*n* = 7)
Bladder Weight (mg)	88.8 ± 12.66	109.5 ± 16.91	108.4 ± 21.08	101.9 ± 17.22	202.0 ± 12.68	160.3 ± 31.81	147.6 ± 24.88	169.3 ± 32.58
Flat lesion								
*None*	4 (100)	8 (100)	7 (100)	8 (100)	0	0	1 (12.5)	0
*Dysplasia*	0	0	0	0	0	0	0	1 (14.3)
*Carcinoma* in situ	0	0	0	0	4 (100)	8 (100)	7 (87.5)	6 (85.7)
Papillary lesion								
*None*	4 (100)	8 (100)	7 (100)	8 (100)	0	0	0	1 (14.3)
*PUNLMP*	0	0	0	0	0	0	1 (12.5)	1 (14.3)
*Non-invasive urothelial carcinoma*	0	0	0	0	3 (75)	7 (87.5)	5 (62.5)	5 (71.4)
*Invasive urothelial carcinoma*	0	0	0	0	1 (25)	1 (12,5)	2 (25)	0
Accompanying squamous metaplasia	0	0	0	0	3 (75)	2 (25)	3 (37.5)	3 (42.9)
Ki-67 Proliferative index (%)	10.0 ± 14.70	3.9 ± 5.08	5.9 ± 6.39	1.6 ± 1.30	30.5 ± 3.11	30.5 ± 15.52	24.9 ± 7.90	20.0 ± 13.59
TNF-α positive inflammatory cells	13.0 ± 4.55	11.6 ± 2.39	11.4 ± 1.40	9.4 ± 1.30	23.3 ± 2.22	30.4 ± 5.01	28.1 ± 4.02	23.4 ± 4.54
Apoptotic index	5.0 ± 4.76	2.9 ± 2.47	4.4 ± 3.36	1.6 ± 1.30	4.8 ± 4.86	11.6 ± 6.44	15.4 ± 5.45	15.6 ± 2.37

*PUNLMP* Papillary urothelial neoplasm of low malignant potential.

Carcinoma in situ was present in the majority of the cancer groups, whereas papillary urothelial carcinomatous lesions were diagnosed in 100%, 100%, 87.5%, and 71.4% of the cancer-only, cancer-cRT, cancer-BNCT-sys, and cancer-BNCT-IV groups, respectively. In addition, invasive urothelial carcinoma was diagnosed at 25% in the cancer-only and cancer-BNCT-syst groups, whereas these rates in cancer-cRT and cancer-BNCT-IV groups were 12.5% and 0%. Detailed information regarding tumour burden, precancerous and carcinomatous lesions of the groups is shown in Table 1.

**Fig. 1:**
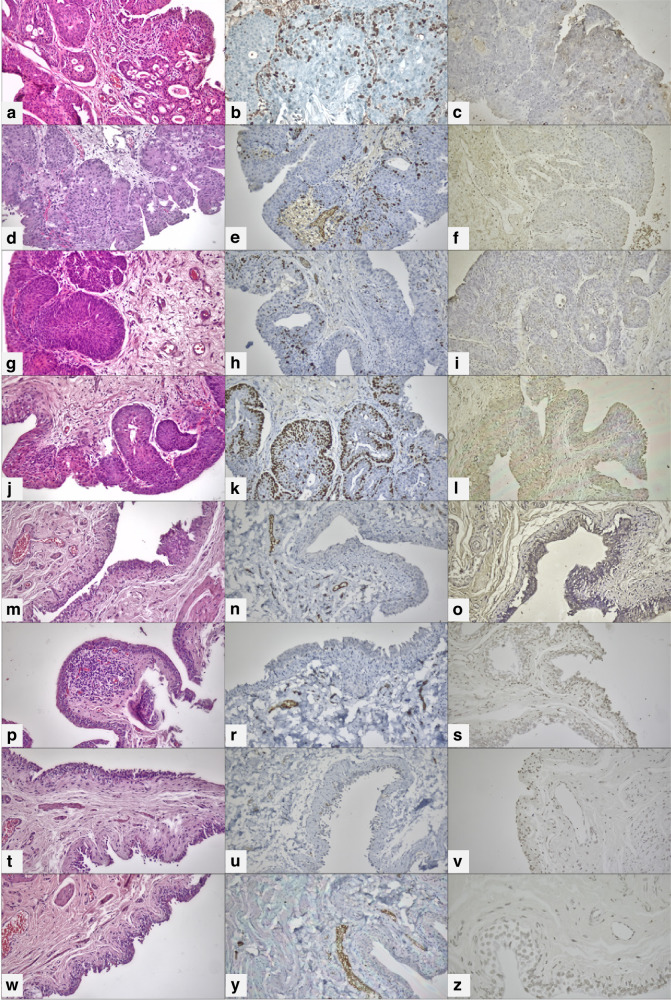
Representative histological and immunohistochemical images of bladder from each study group. From left to right: Hematoxylin & Eosin (H&E), Ki-67, and TUNEL staining (original magnification, ×200). (**A**–**C**) Cancer group; (**D**–**F**) Cancer-cRT group; (**G**–**I**) Cancer-BNCT-IV group; (**J**–**L**) Cancer-BNCT-sys group; (**M**–**O**) Control group; (**P**–**S**) Control-cRT group; (**T**–**V**) Control-BNCT-IV group; (**W**–**Z**) Control-BNCT-sys group.

**Fig. 2:**
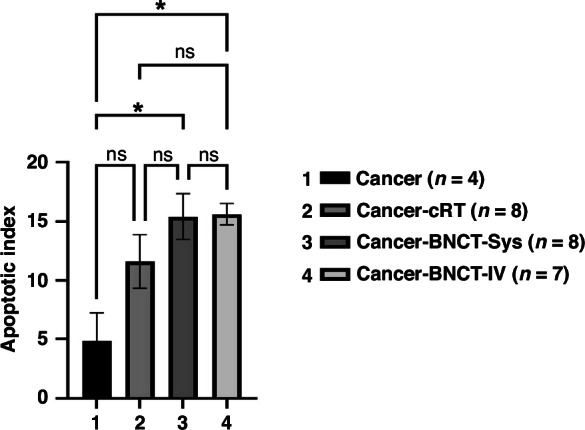
Comparison of apoptotic cell numbers between cancer groups. “*” represents a significant *p* value. *ns: not significant*.

Proliferation indexes and apoptotic indexes with microphotos of H&E stained sections from groups are shown in Fig. 1. Unsurprisingly, the Ki-67 proliferative indexes were significantly higher in the cancer groups than in control groups (F [1,46] = 58.81, *p* < 0.001, η² = 0.561). Among control groups, treatment type did not significantly affect Ki-67 levels (F [3,23] = 1.43, *p* = 0.259). In urothelial carcinogenesis induced groups, Ki-67 values did not differ significantly across treatment arms (F [3,23] = 1.19, *p* = 0.337). Although the Cancer-BNCT-IV group showed the lowest mean Ki-67 index (mean = 20.0%), post hoc comparisons revealed that this reduction was not statistically significant (*p* > 0.05). Apoptotic indexes were significantly higher in urothelial carcinogenesis induced groups compared with non-induced controls (F [1,46] = 50.330, *p* < 0.001, η² = 0.522, Table 1). Among control groups, no significant differences in apoptotic indexes were found across treatment groups (F [3,23] = 1.779, *p* = 0.179), although numerically lower values were observed in the Control-BNCT-IV group. In urothelial carcinogenesis induced groups, apoptotic indexes differed significantly between treatment groups (F [3,23] = 4.799, *p* = 0.010). Post hoc analysis showed that both BNCT-Sys and BNCT-IV groups had significantly higher apoptotic indexes compared with the Cancer-only group (*p* = 0.015 and *p* = 0.016, respectively), while no significant difference was found between the Cancer-only and Cancer-cRT- groups (*p* = 0.232, Fig. 2).

### The examination of pro-inflammatory findings in paravesical tissues and bladder after radiation therapies

The incidence of moderate-severe pro-inflammatory response in the colons of radiation treated groups were 37.5% (6/16) for cRT, 33.3% (5/15) for BNCT-syst, and 6.6% (1/15) for BNCT-IV. Similarly, for uterine tissues, these values were 25% (4/16) for cRT, 33.3% (5/15) for BNCT-syst, and 6.6% (1/15) for BNCT-IV. Moderate-severe pro-inflammatory distribution in the abdominal anterior wall was seen in only 1 (6.6%) animal from BNCT-syst treated groups. In the bladder, the incidence of moderate to severe inflammation was also lower in the BNCT-IV group (20.0%) compared with the cRT (50.0%) and BNCT-Sys (33.3%) groups (Table 2). Microphotographs of paravesical and bladder tissues indicating moderate-severe inflammation are shown in Supplementary Fig. 9.

**Table 2:** Presence of moderate to severe inflammation after irradiation in the bladder and surrounding tissues.

Groups	cRT (Control + Cancer) (*n* = 16)	BNCT-Sys (Control + Cancer) (*n* = 15)	BNCT-IV (Control + Cancer) (*n* = 15)
Bladder; *n* (%)	8 (50)	5 (33.3)	3 (20.0)
Anterior abdominal wall; *n* (%)	0	1 (6.7)	0
Uterus; *n* (%)	4 (25)	5 (33.3)	1 (6.6)
Colon; *n* (%)	6 (37.5)	5 (33.3)	1 (6.6)

### Comparative bladder TNF-α expression levels among cancer untreated and cancer-treated groups using immunohistochemistry and western blotting

**Fig. 3:**
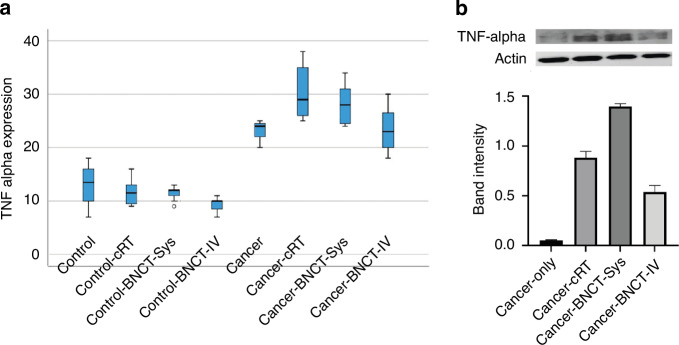
Comparative analysis of TNF-alpha expression in bladder tissues. **a** Quantative analysis of TNF-α positive inflammatory cells among all groups using Immunohistochemistry. **b** Western blot experiments show TNF-α expression levels in bladder tissues among cancer groups.

To further analyse the pro-inflammatory findings in bladder tissues, TNF-α expression was investigated. First, IHC examination showed that TNF-α positive inflammatory cells were relatively more common in cancer groups compared with the control groups (Supplementary Fig. 10). Additionally (Fig. 3a), TNF-α positive inflammatory cells were significantly more abundant in urothelial carcinogenesis induced groups compared with the control groups (F [1,46] = 231.109, *p* < 0.001, η²=0.834). Among urothelial carcinogenesis induced groups, TNF-α positive inflammatory cells differed significantly between treatment arms (F [3,23] = 4.418, *p* = 0.014). Post hoc analysis showed that TNF-α positive inflammatory cells in the cancer-BNCT-Sys group were higher than in the Cancer-only group, although this difference was not statistically significant (*p* > 0.05). The Cancer-BNCT-IV group exhibited similar TNF-α positive inflammatory cells to the Cancer-only group (*p* > 0.05), and significantly lower levels than both the Cancer-cRT (*p* = 0.029) and Cancer-BNCT-Sys groups (*p* = 0.007).

Moreover, Western blot analysis revealed significant differences in bladder TNF-α expression levels among the experimental groups (Welch’s ANOVA, F [3,1.72] = 970.679, *p* = 0.002). TNF-α levels were higher in the cancer-treated groups compared with the Cancer-only group, and relatively lower TNF-α levels were observed in the BNCT-IV group compared with the other treatment groups (Fig. 3b).

## Discussion

Radiation therapy is generally used in three forms, external beam radiotherapy (EBRT), brachytherapy and systemic radionuclide therapy. EBRT and brachytherapy are treatment methods based on the macroscopic irradiation of tissue aided by the imaging modalities with magnetic resonance imaging or computerised tomography, while radionuclide therapy is microscopic cellular-level radiotherapy performed by directing radioisotopes to the target cell through a vein [5]. An example of this micro-level radiotherapy is BNCT, a treatment method based on the toxic effect of linear energy resulting from nuclear reactions at the cellular level in tumour cells. Radiation therapy is used especially in the muscle-invasive stage of localised bladder cancer as a part of multimodal treatment, in selected cases for palliative purposes in non-muscle-invasive bladder cancer and also in metastatic disease, usually in the form of EBRT. While EBRT has a tumoricidal effect in bladder cancer, it can also cause deterioration in the function of a dynamic and functional organ, such as radiation cystitis and incontinence. Therefore, the efficacy of radiotherapy at a cellular level and specific to the bladder tumour may provide advantages, such as better antitumoral effect and may also cause fewer side effects related to radiotherapy in non-tumoral areas of the bladder. Thus, BNCT may be a promising treatment method in bladder cancer. In mouse models where MBT2 bladder cancer cells were injected subcutaneously, a reduction in the size of the tumour tissue was reported following BNCT [18]. However, this tumour model was a subcutaneous cancer model and does not reflect the bladder, which can be interpreted as a limitation of that study. We believe that a demonstration of both antitumoral effect and improved side effect profile related to BNCT in the bladder microenvironment after treatment in an orthotopic bladder cancer model may be more informative. The present study was the first to evaluate the antitumoral and side effect profile of BNCT following systemic or intravesical BPA application in an experimental orthotopic bladder cancer model. In addition, the side effect profiles and antitumoral effect of BNCT were compared with 6 Gy cRT applied as EBRT. Although the rate of persistent tumours was high in all cancer groups, a decrease of approximately 15-30% in tumour burden was observed in animals that received BNCT and cRT. Despite this similar rate of decrease in tumour burden in the animals receiving cRT and BNCT, particularly in the groups treated with a BNCT intravesical approach, the lower Ki-67 expression levels and higher apoptotic cell rates compared to animals that did not receive any radiotherapy, combined with the finding of an absence of papillary urothelial carcinoma in 28%, although not significant, are the most remarkable antitumoral findings from our study. The lower pro-inflammatory side effect profile in bladder and perivesical tissues accompanying this antitumor activity are also notable findings in the Cancer+BNCT-IV group.

Reactor-based neutron providers and BNCT have more often been tested in tumours close to the skin. This is because the effect of thermal neutron beams decrease as they go deeper into the tissues. Epithermal neutrons may penetrate deeper into tissues [6]. Malignant melanoma, brain tumours and head and neck tumours are malignancies that have been investigated with BNCT and are close to the skin [19–21]. In the present study, CT showed that the bladder tissues of the rats were generally less than 2 cm deep, and the treatment efficacy of BNCT generated with thermal neutrons in bladder tissue that is close to the skin at this level was compared with conventional radiotherapy. Despite similar antitumoral activity, less inflammation was detected in the bladder tissue and perivesical organs (colon and uterus), especially in animals receiving BNCT in the intravesical group. We suggest that better results may be obtained when BNCT activity is investigated in human bladder cancer tissue using epithermal neutrons that provide deeper tissue penetration via accelerator-based neutron providers instead of thermal neutrons as used in our study.

In BNCT, the low accumulation of boron molecules in tumour cells is one of the main problems that reduces treatment efficacy. Recently, in order to overcome this issue, third generation boron compounds are currently being developed and being used in BNCT studies instead of second generation boron compounds, such as BPA (used in the present study) and borocaptate sodium (BSH) [22]. In the present study, intravesical BPA dissolved in DMSO exhibited more (up to six-fold) boron accumulation in bladder tissue compared to systemic BPA application. Especially in the intravesical BNCT groups, 28% totally absence of papillary urothelial carcinoma and the lower Ki-67 expression may be associated with this greater boron accumulation. Although boron accumulation in bladder tissues was less in our study than previously reported [23], the histopathological findings and side effect profiles in our BNCT treatment groups were no worse than cRT. In the future, third generation molecules that may provide greater levels of boron accumulation in target tissues may be a more effective means of treatment in BNCT for bladder cancer.

In the present study, the tumour burden reducing effect of 6 Gy EBRT was achieved using a limited number of thermal neutron beams (4 × 10^5^ cm^−2^ s^−2^) with one hour of irradiation. Germany and Italy are other countries where BNCT studies have been conducted using TRIGA MARK II reactors [24, 25]. One of these reactors is also located in Istanbul Technical University. Instead of an old neutron provider facility, such as the TRIGA MARK II reactor, studies may be conducted with accelerator-based neutron providers that may yield a higher proportion of thermal neutrons and faster epithermal neutrons, allowing deeper tissue penetration and providing better treatment outcomes for cases of human bladder cancer. This may lead to more boron neutron capture fusion reactions and thus result in a more effective treatment.

In our experiments, even at 6 Gy EBRT, no complete pathological response was seen in the cRT group, and the tumour burden was reduced by only 20%. Although this tumour burden-diminishing effect was at a similar rate in the BNCT arms (systemic or intravesical), papillary urothelial cancerous lesions were almost 30% less prevalent in the intravesical arm. Furthermore, the decrease in proinflammatory TNF-α expression in bladder tissue after neutron irradiation following intravesical BPA application compared with the other cancer+cRT and systemic BNCT groups and the decrease in the number of inflammatory cells may be explained by greater effect of intravesical BNCT on tumour cells, while having less inflammatory influence on normal bladder tissue. We acknowledge that the superior inflammatory profile in bladder tissues observed for the intravesical BNCT group may be partly influenced by the vehicle used for intravesical delivery. DMSO has well-recognised anti-inflammatory and analgesic properties in the urinary bladder and is used clinically as an intravesical therapy for interstitial cystitis/bladder pain syndrome [10, 26]. Mechanistically, DMSO has been shown to modulate inflammatory signalling (including NF-κB/MAPK-related pathways) and to attenuate pro-inflammatory cytokine responses such as TNF-α in experimental systems, which could confound attribution of reduced inflammation solely to the spatial precision of BNCT [27–30]. Accordingly, future studies should validate whether the improved inflammatory profile persists with alternative intravesical vehicles/solvents (or solvent-free formulations), ideally including a vehicle-only control arm. Differences observed between immunohistochemical TNF-α staining and Western blot analyses may reflect methodological distinctions, including the assessment of spatially localised inflammatory cells by immunohistochemistry versus total protein levels measured in pooled tissue homogenates by Western blotting.

The limiting factors of our study are that boron appeared to accumulate less in our bladder tissues compared to the literature and the number of neutron beams was relatively lower. In the future, these limiting factors can be eliminated with accelerator-based neutron providers and/or newer third generation boron source molecules. It is hoped that more effective BNCT can be developed in the future for human bladder cancer. Finally, it is known that radiotherapy can cause secondary malignancies in the long term. Therefore, long-term follow-up studies are needed to determine whether BNCT may change the rate of secondary malignancy development.

In conclusion, the present study is the first to evaluate the antitumoral and side effect profile of BNCT following systemic or intravesical boron application in an orthotopic bladder cancer model and to compare these effects with cRT. These results showed that systemic or intravesical BNCT achieved a partial tumour burden reduction comparable to cRT, whereas intravesical administration of BPA for BNCT treatment was associated with a more favourable inflammatory/side effect profile, which may be partly influenced by the intravesical vehicle (DMSO). Therefore, BNCT using intravesical boron may represent a potential radiotherapeutic option for bladder cancer in the future; however, further studies employing alternative intravesical formulations and appropriate vehicle controls are warranted, particularly in the context of emerging boron carrier molecules and more effective neutron sources.

## Supplementary information

supplementary information file

## Data Availability

The datasets generated during and/or analysed during the current study are available from the corresponding author on reasonable request.
