# Supplementary material for: Boron neutron capture therapy (BNCT) for experimental bladder cancer: systemic or intravesical approach
Source: Br J Cancer. 2026 Apr 17;135(2):223–31. doi: 10.1038/s41416-026-03418-w (PMC13310855; doi:10.1038/s41416-026-03418-w)
Supplement: Supplementary file 1 — supplementary information file [file 41416_2026_3418_MOESM1_ESM.pdf]

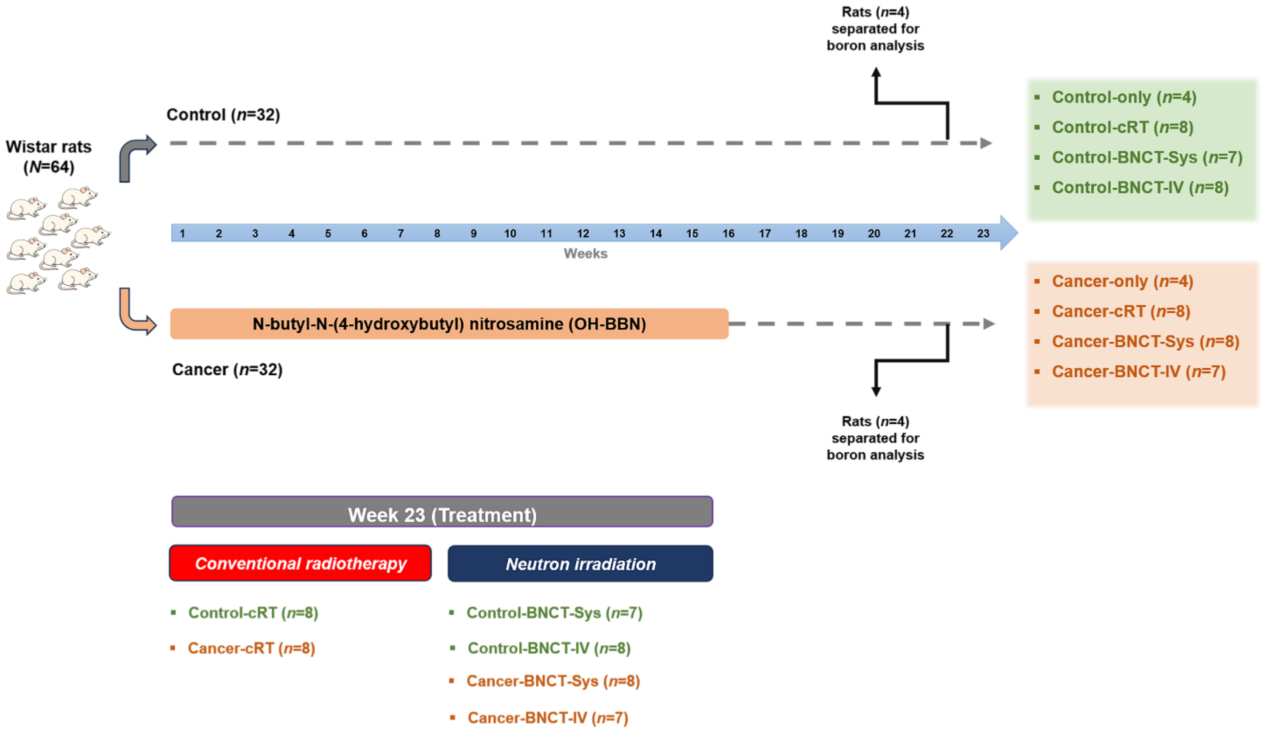

Supp. Figure 1.

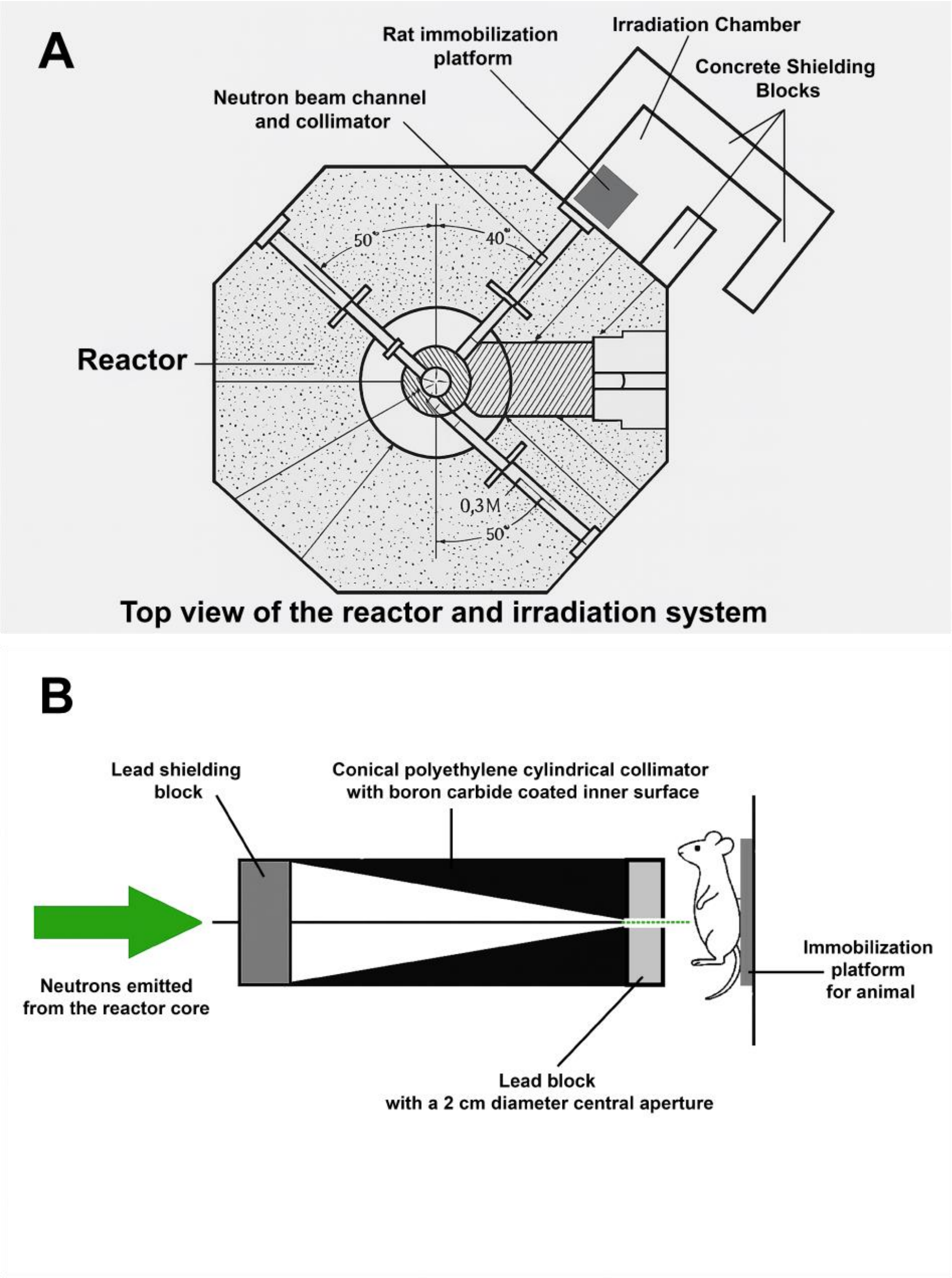

Supp. Figure 2.

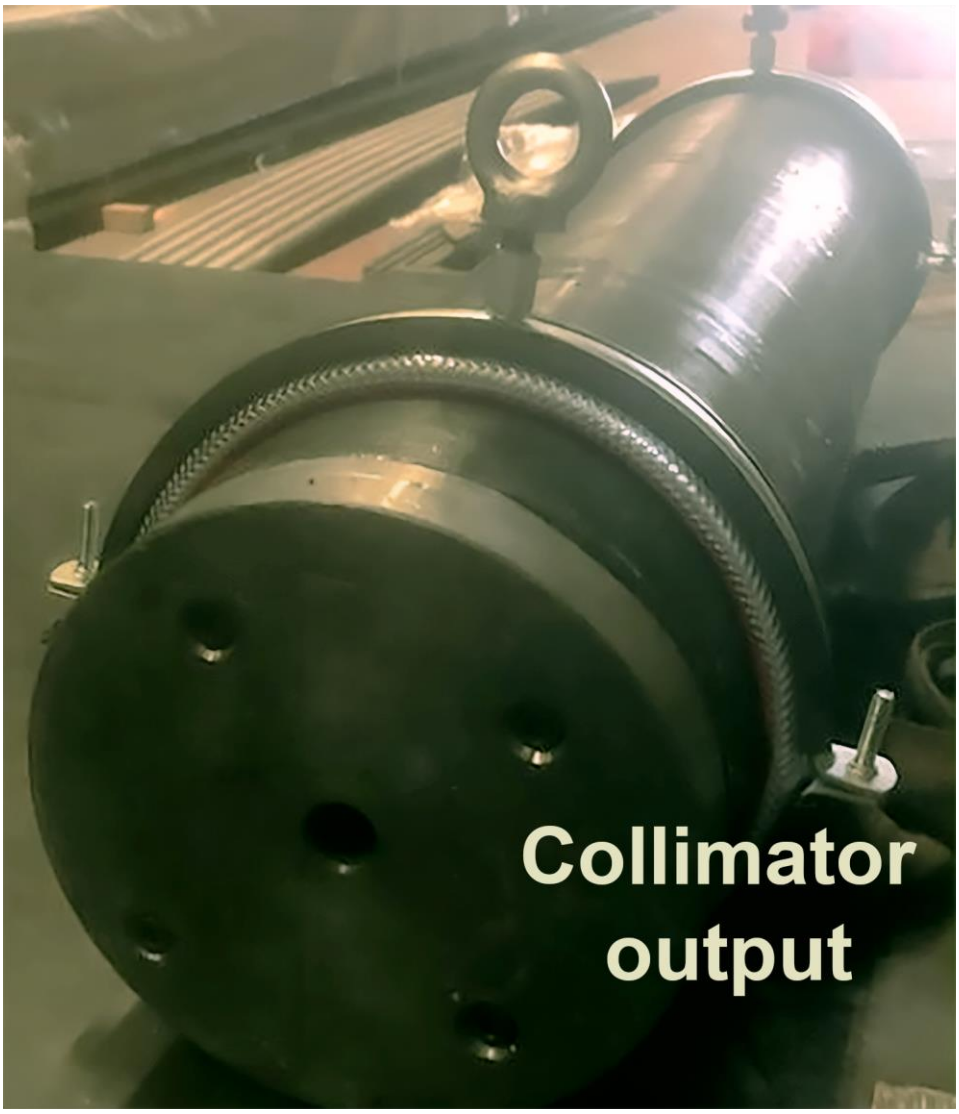

Supp. Figure 3.

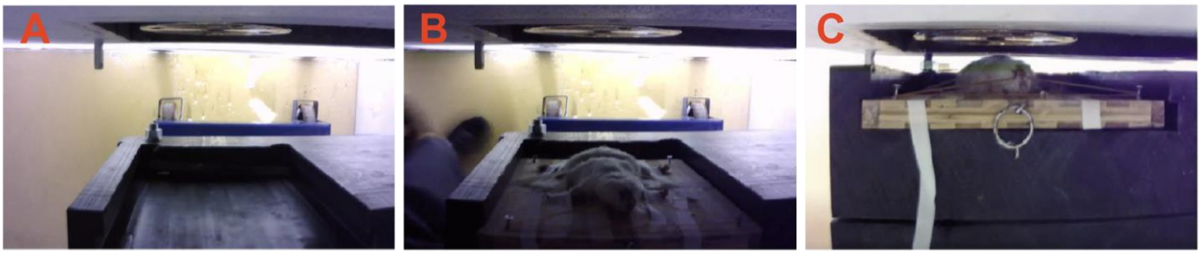

Supp. Figure 4.

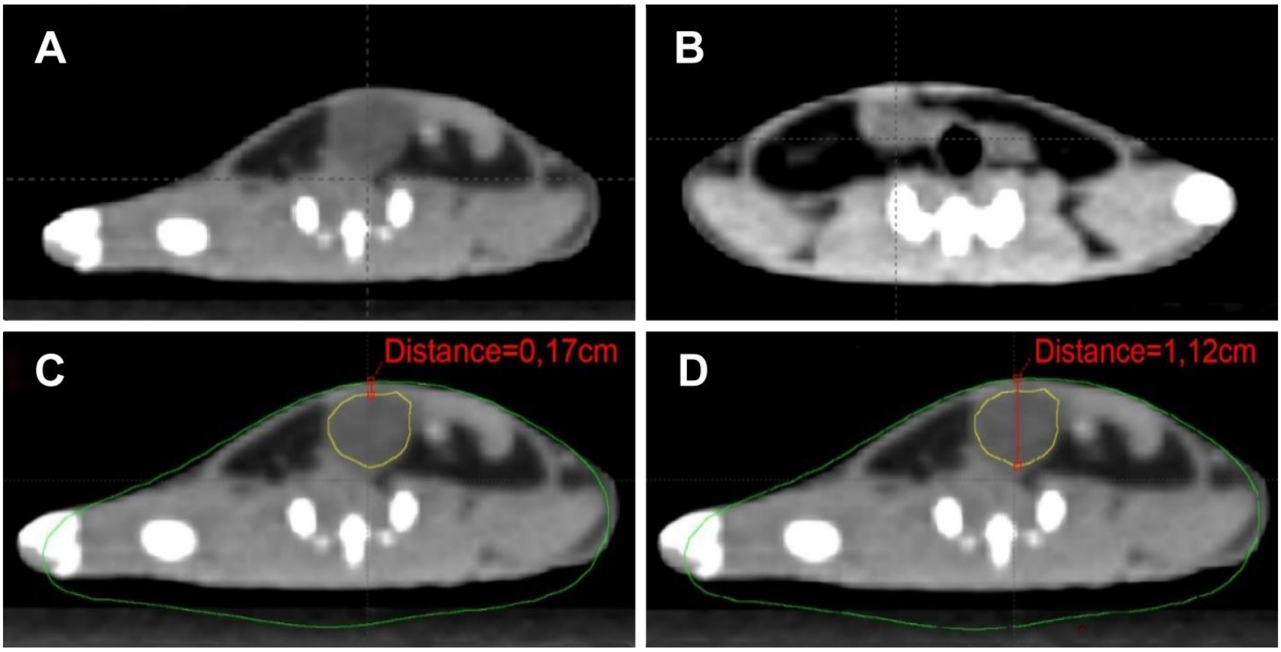

Supp. Figure 5.

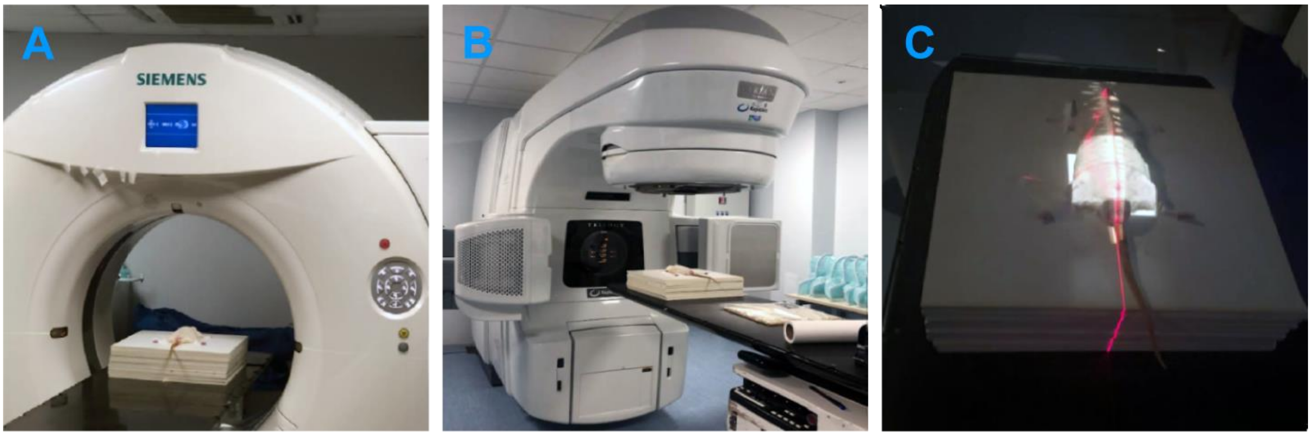

Supp. Figure 6.

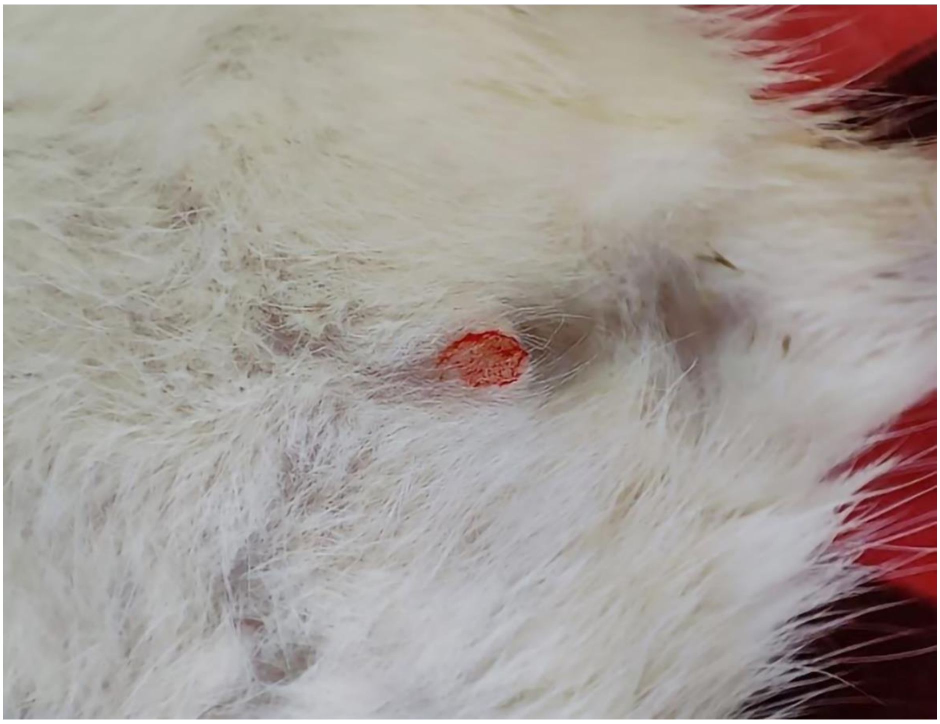

Supp. Figure 7.

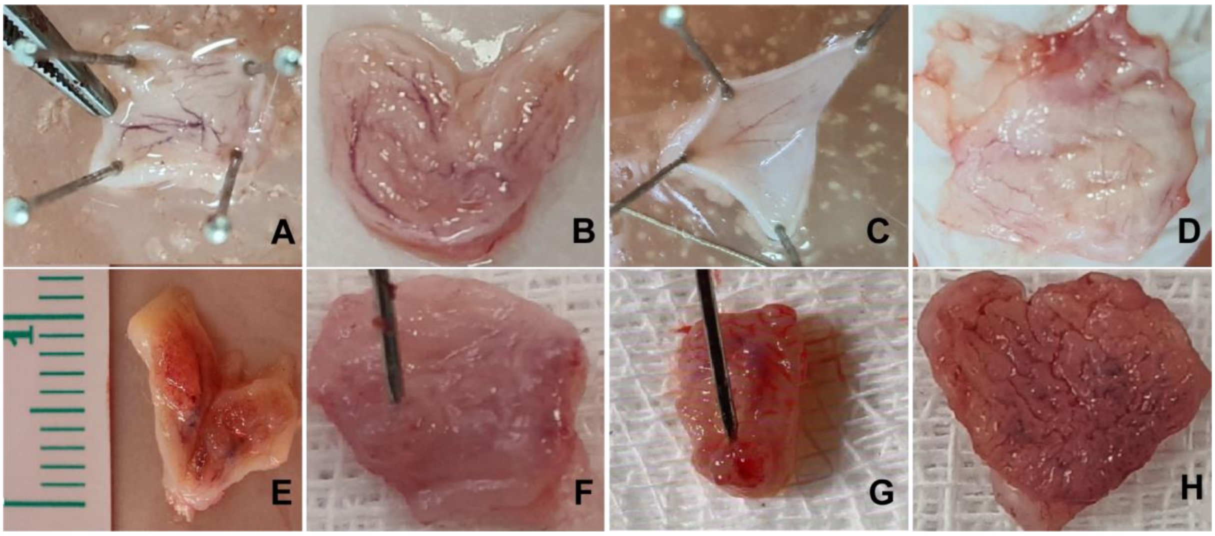

Supp. Figure 8.

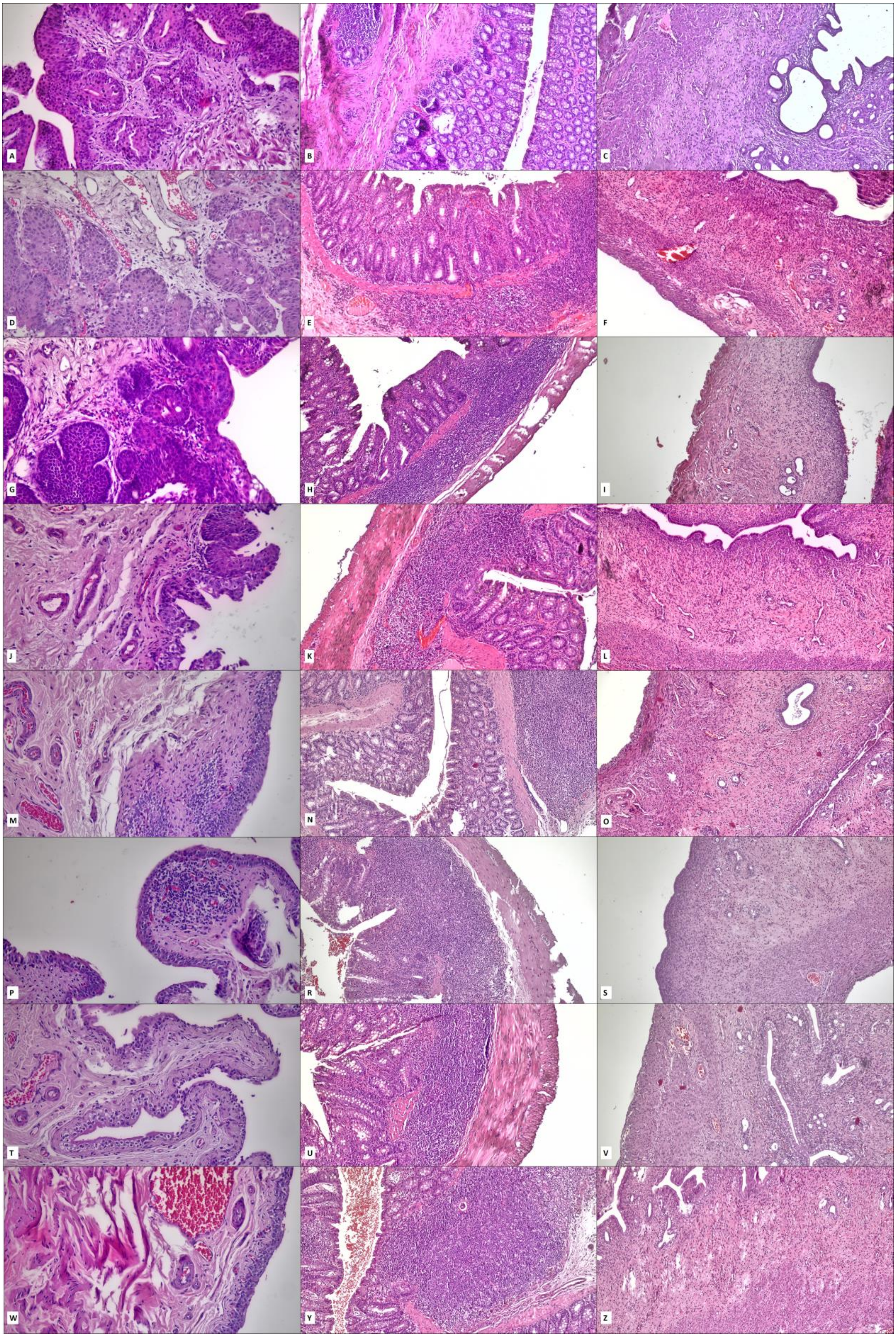

Supp. Figure 9.

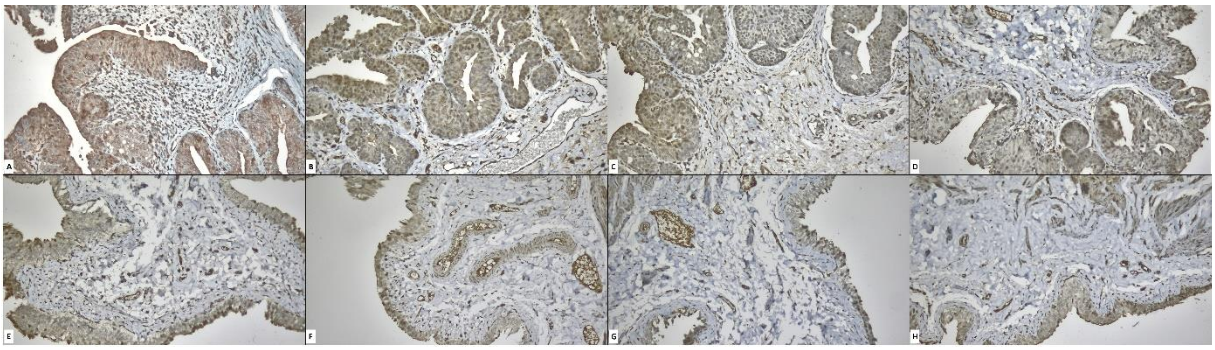

Supp. Figure 10.

Supplementary Figures' legends

Supp. Figure 1. Experimental design of the study.

Supp. Figure 2. Neutron irradiation platform at ITU-MARK TRIGA II reactor. **A)** Top view of the reactor and irradiation chamber. **B)** Rat immobilization set-up**.**

Supp. Figure 3. View of the colimator output of the reactor.

Supp. Figure 4. Top view of the animal aligned with the collimator output. **A)** Polyethylene case**. B)** A sedated animal fixed in a polyethylene case. **C)** The image of the rat fixed towards the collimator outlet.

Supp. Figure 5. Axial section view of the rat bladder. **A)** Image of the rat bladder with noticeable regular borders from the control group. **B)** Image of a rat bladder that received cancer induction with a notable increase in tumor thickness on the right lateral wall. **C)** Calculation of the distance between the anterior wall of the rat bladder and the skin. **D)** Calculation of the distance between the posterior wall of the rat bladder and the skin.

Supp. Figure 6. The determination of the treatment core of the rat bladder using computed tomography-guided imaging (**A-C**).

Supp. Figure 7. Genital image of an animal with macroscopic hematuria.

Supp. Figure 8. Macroscopic images of the luminal surfaces of the bladders of all groups. Needles show obvious bladder tumors. **A)** from Control-only group. **B)** from Control-cRT group. **C)** from Control-BNCT-sys group. **D)** from Control-BNCT-IV group. **E)** from Cancer-only group. **F)** from Cancer-cRT group. **G)** from Cancer-BNCT-sys group. **H)** from Cancer-BNCT-IV group.

Supp. Figure 9. Representative histological images of bladder (left column), colon (middle column), and uterus (right column) tissues from each study group stained with Hematoxylin & Eosin (H&E), original magnification ×100. (A–C) Cancer group; (D–F) Cancer-cRT group; (G–I) Cancer-BNCT-IV group; (J–L) Cancer-BNCT-Sys group; (M–O) Control group; (P–S) Control-cRT group; (T–V) Control-BNCT-IV group; (W–Z) Control-BNCT-Sys group.

Supp. Figure 10. Immunohistochemical staining of TNF-α expression in bladder tissues across all experimental groups. A. Cancer group (×200); B. Cancer-cRT group (×200); C. Cancer-BNCT-IV group (×200); D. Cancer-BNCT-Sys group (×200); E. Control group (×200); F. Control-cRT group (×100); G. Control-BNCT-IV group (×100); H. Control-

BNCT-Sys group (×100). Positive TNF-α staining was seen primarily in inflammatory cells, with reduced intensity observed in the intravesical BNCT group compared to others.
